# Modeling of Integrated Hollow-Fiber Solar-Powered VMD Modules for Desalination for a Better Understanding and Management of Heat Flows

**DOI:** 10.3390/membranes14020050

**Published:** 2024-02-11

**Authors:** Gina Alfonso, Stéphanie Laborie, Corinne Cabassud

**Affiliations:** TBI, Université de Toulouse, CNRS, INRAE, INSA, 135 Avenue de Rangueil, Cedex 4, F-31077 Toulouse, France

**Keywords:** solar-assisted desalination, integrated hollow fiber module, vacuum membrane distillation, energy analysis, modeling and simulation

## Abstract

The direct integration of membrane distillation and solar energy collection in a single module is a promising technology for autonomous seawater desalination in remote regions; however, the modeling and design of such modules are challenging because of the coupling of the radial and longitudinal heat and mass transfers. In a previous study, we provided as a first modeling approach a hollow fiber solar collector vacuum membrane distillation (VMD) module, considering a constant temperature at the shell side and a pure water feed. Here, a full model is developed to describe the coupled effects of the solar collector and a hollow fiber VMD module operating in an outside/in mode with saline water. The model considers all the main phenomena (membrane distillation, temperature and concentration polarization, absorption of solar radiation and energy balances over the solar collector, radial and longitudinal heat and mass transfer, seawater properties, and more than 30 variables). Applied to simulate the behavior of a semi-industrial-scale module, it allows the influence of solar radiation on the performance/limits of the integrated module to be discussed based on the radial and longitudinal profiles and heat flows. The model can be used to identify key points in the module design to better utilize solar radiation and manage heat flows.

## 1. Introduction

Desalination via vacuum membrane distillation (VMD), and its direct coupling with renewable energy such as solar energy, has been continuously improved over the last decade [1,2,3,4,5,6,7,8] because VMD is a process based on thermal energy [9] and can be used to treat hypersaline streams [10,11]. Méricq et al. [1] published one of the first studies on the direct integration of a VMD and a solar collector, leading to a solar collector vacuum membrane distillation (SC-VMD) module in which the feed is heated within the same module where the VMD process occurs. This module behaves simultaneously as both a membrane contactor for distillation and as a solar collector, decreasing the thermal losses compared with systems in which the membrane module is separated from the solar collector. In addition, integrated SC-VMD modules provide compactness and modularity to the system unlike conventional distillation [12].

During the last 5 years, research on integrated membrane distillation modules coupled with solar energy has advanced via the application of innovative concepts and technologies. The modeling and optimization of a solar flat plate collector VMD module has revealed that a membrane surface area of 3 m^2^ is capable of producing 96 L of fresh water per day [2,4], demonstrating the potential of such an integrated system to supply fresh water to small communities in remote areas.

Considering the higher surface-to-volume ratio of hollow fibers (HFs) compared with flat sheet membranes [13], this geometry is of interest to generate compact modules. Consequently, experimental and modeling studies have focused on the design and testing of HFs introduced into conventional solar collectors at the lab scale [3,5,7]. These studies show a lack of flexibility in the module design and indicate the importance of considering the radial temperature profile between the solar collector and the membrane to avoid overestimating the permeate production.

**Figure 1:**
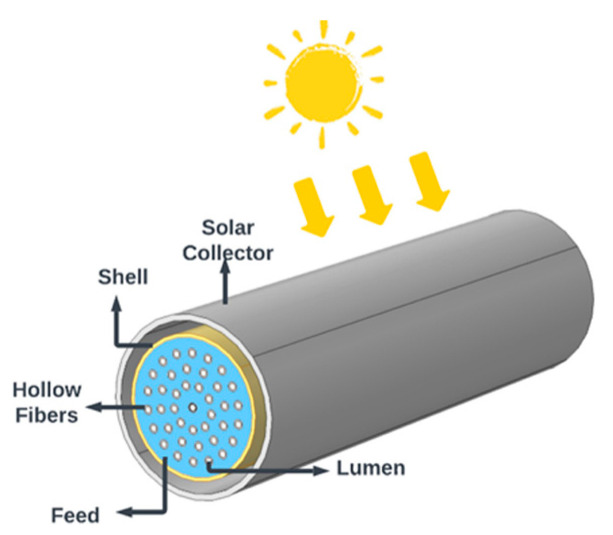
Schematic of the hollow fiber solar collector vacuum membrane distillation (HF-SC-VMD) module taken from Ref. [8].

Subsequently, an innovative solar collector directly integrating an HF bundle acting as a single device was proposed by Alfonso et al. [8]. The proposed integration supplies solar energy via the external cylindrical module surface to heat the feed surrounding the fibers operating in an outside/in mode as illustrated in Figure 1. To achieve consequential vaporization, the solar collector must be capable of absorbing the bulk of the solar radiation, concentrating it, and transferring it to the fluid. This transfer depends on multiple variables, including solar radiation (daily and seasonal), location, module orientation, and environmental conditions [8].

The feasibility of the hollow fiber solar collector vacuum membrane distillation (HF-SC-VMD) module was validated in Ref. [8] via a first approximation achieved by fixing a constant temperature at the shell wall (*T_wall_*) and by feeding the module with pure water. Using this approach, it was demonstrated that a module with a membrane area of 2.1 m^2^ could produce between 2.1 kg/h/m^2^ and 18 kg/h/m^2^ (from 4.46 L/h to 37.4 L/h) of fresh water with a *T_wall_* of 70 °C depending on the feed inlet temperature and the operating conditions. These permeate flow rates demonstrate the maximum production generated for this module. However, other phenomena may decrease its productivity, such as heat losses through the solar collector and concentration polarization at the module feed side, depending on the feed salinity and the hydrodynamics.

Modeling the heat and energy transfer from solar heat radiation to the fluid involves considering the following phenomena: energy absorption, heat transfer toward the fluid, and heat losses to the environment. These heat exchanges determine the actual fluid temperature at the shell wall and the local permeate flux, which may differ from the values estimated when considering a constant *T_wall_*.

Therefore, the main objectives of this study are twofold. The first objective is to develop a model describing the behavior of an HF-SC-VMD module, which will then be employed to optimize the design of this innovative module. The model considers more than 30 variables describing the location, module dimensions, material properties, solar collector properties, membrane permeability, seawater salinity, and operating conditions. The second objective is to use the model to estimate the performance of a semi-industrial-scale module, analyze the influence of solar energy on the temperature and permeate flow rate production of the HF-SC-VMD module, and compare the model results with those achieved in Ref. [8] using a general approach (pure water and a fixed *T_wall_*).

## 2. Principle of the HF-SC-VMD Module

The current HF-SC-VMD module is based on the module concept defined in Ref. [8]; however, here, the solar collector components and their properties are considered to more precisely estimate the module performance, to describe a feasible solar collector, and to consider variations in the transferred energy according to the fluid characteristics, operating conditions, solar collector dimensions, and materials.

**Figure 2:**
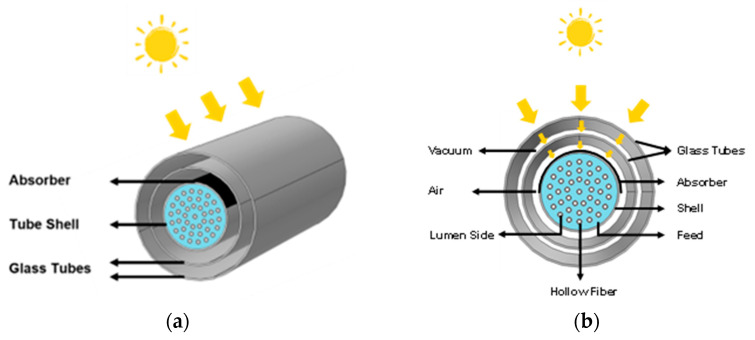
(**a**) Schematic of an HF-SC-VMD module with two glass tubes. (**b**) Radial profile of the module.

The solar collector is based on two concentric glass tubes, as shown in Figure 2. A vacuum is present between the two tubes, as represented by the white space between the tubes in Figure 2b. This configuration is employed to decrease heat loss to the environment and to simultaneously ensure that most of the solar radiation is transmitted to the absorber. This design was selected because of the technical feasibility of using a vacuum between the VMD shell side and the environment. The space between the shell and the internal glass tube is occupied by air. In addition, we assume that an absorber layer is placed over the upper side of the module shell (representing 50% of the shell area) to better absorb incident solar radiation. That is, half of the tube shell surface receives solar radiation from the top of the module. This is illustrated by the black surface over the shell in Figure 2.

The heat transfer in the module depends on various phenomena. To understand the heat transfer, it is important to define several parameters. First, the solar radiation arriving at the Earth’s surface is defined by extraterrestrial solar radiation (*G_on_*), which depends on the day and the solar constant (*G_SC_*).

**Figure 3:**
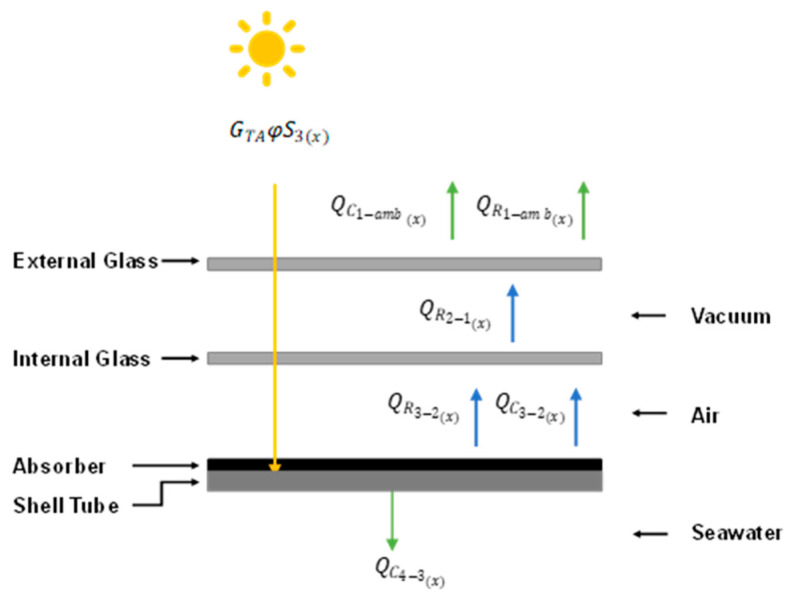
Energy flows in the concentric layers of the solar collector in an HF-SC-VMD module. Here, the subscripts represent the (1) external glass layer, (2) internal glass layer, (3) shell, and (4) bulk feed near the shell.

Figure 3 illustrates that the solar radiation is absorbed by the absorber and is defined as $G_{TA}\varphi S_{3(x)}$, where $G_{TA}$ is the absorbed solar radiation considering the reflection and absorbance losses resulting from the glass layers, φ is the portion of the shell covered by the absorber, and $S_{3(x)}$ is the total shell surface. Part of this energy flow is transferred to the fluid (*Q_C_*_4-3_), while the rest is lost and transferred from the absorber to the outer part of the solar collector via radiation and convection. The energy balance is specified in the following sections by defining the different energy flows through the solar collector. These balances are coupled to the HF-SC-VMD module to ultimately estimate the fluid temperature profile and permeate production.

## 3. Modeling Methodology

Different inlet variables are involved in the VMD process and may influence the performance of the HF-SC-VMD module; these include the radial and longitudinal temperature profiles and the permeate flux and flow rate. Important variables also include environmental conditions, solar collector dimensions, material properties, membrane characteristics, and operating conditions.

The finite volume difference method is used for the 2D modeling based on the inlet variables. The module is discretized radially and longitudinally, generating a mesh. The energy and mass balances are established in each unit element and then solved. The modeling methodology is described in detail in Ref. [8].

In this study, the equations for determining the solar radiation based on the day and location, as well as the equations describing the absorption of solar radiation, are integrated considering the material properties and the heat transfer from the solar collector to the fluid and environment. The full model developed here includes the energy balances to describe the behavior of the solar collector (described in Section 4) and the equations describing the behavior of the VMD on the shell side based on Equations (1)–(6) introduced in Ref. [8]. In addition, we consider the feed salt concentration because of its impact on the physico-chemical properties of the feed, liquid–vapor equilibrium, and concentration polarization, which directly affect the permeate flow rate produced by the HF-SC-VMD module [14].

The general mass and energy balances for each node are expressed as

(1) $$F_{\left(r,x-1\right)}=F_{\left(r,x\right)}+F_{p(r,x)}$$

(2) $$Q_{r_{\left(r-1,x\right)}}+Q_{x_{\left(r,x-1\right)}}=Q_{r_{\left(r,x\right)}}+Q_{x_{\left(r,x\right)}}+Q_{p_{\left(r,x\right)}}$$

The radial heat conduction at the node inlet ($Q_{r_{\left(r-1,x\right)}}$) and at the outlet ($Q_{r_{\left(r,x\right)}}$) are expressed as

(2a) $$Q_{r_{\left(r-1,x\right)}}=-k_{r-1,x}S_{r-1,x}\frac{\partial T}{\partial r}=-k_{r-1,x}S_{r-1,x}\frac{T_{r-1,x}-T_{r,x}}{dr}$$

(2b) $$Q_{r_{\left(r,x\right)}}=-k_{r,x}S_{r,x}\frac{\partial T}{\partial r}=-k_{r,x}S_{r,x}\frac{T_{r,x}-T_{r+dr,x}}{dr}$$

The energy transferred as a result of the feed flow rate is

(2c) $$Q_{x_{\left(r,x-1\right)}}=C_{p(r,x-1)}F_{\left(r,x-1\right)}T_{\left(r,x-1\right)}$$

(2d) $$Q_{x_{\left(r,x\right)}}=C_{p(r,x)}F_{\left(r,x\right)}T_{\left(r,x\right)}$$

The permeate flux is calculated based on the Knudsen diffusion transfer mechanism, which is predominant in the VMD process, where the collisions between the molecules and the membrane wall are dominant [9,15]. The membrane Knudsen permeability (*K_m_*,*_Tref_*) is determined experimentally.

(3) $$J_{p_{r,x}}=\frac{K_{m,T_{ref}}}{\sqrt{M_{H2O}}}\sqrt{\frac{T_{ref}}{T_{m_{\left(r,x\right)}}}}(P_{m_{\left(r,x\right)}}^{*}-P_{p})$$

In this process, and according to the operating conditions, temperature polarization can occur [16]. This phenomenon near the membrane surface is described by a heat transfer coefficient and the temperature gradient between the bulk and the membrane [10].

(4) $$Q_{p_{\left(r,x\right)}}=h_{\left(T\right)}\left(T_{\left(r,x\right)}-T_{m\left(r,x\right)}\right)S_{ext,r}=J_{p_{\left(r,x\right)}}∆H_{v_{\left(r,x\right)}}S_{int,r}$$

(5) $$h_{\left(T\right)}=\frac{Nu\times k_{m}}{D_{H}}$$

(6) $$Nu=0.042\cdot {Re}^{0.59}{Pr}^{0.33}$$

The heat transfer coefficient is calculated based on Equation (6). This correlation is specific to hollow fiber membranes in an outside/in configuration [17]. In addition, the bulk salt concentration ($C_{\left(r,x\right)}$) is considered to determine the seawater properties and the module’s general and local salt balance.

(7) $$F_{\left(r,x-dx\right)}C_{\left(r,x-dx\right)}=F_{\left(r,x\right)}C_{\left(r,x\right)}$$

The simulated seawater consists of NaCl in pure water. Finally, using the heat and mass transfer correlation analogy, the mass transfer coefficient representing the transfer over the boundary layer near the membrane surface can be calculated ($C_{m(r,x)}$). Equation (8) is employed, assuming that the salt concentration in the permeate is zero [18].

(8) $$J_{\left(r,x\right)}=\rho_{\left(r,x\right)}k_{\left(r,x\right)}\log\left(\frac{C_{m(r,x)}}{C_{\left(r,x\right)}}\right)$$

The film model equation relates the concentrations on the feed side, the mass transfer coefficient ($k_{\left(r,x\right)}$), and the seawater density ($\rho_{\left(r,x\right)}$). $k_{\left(r,x\right)}$ is calculated based on an analogy of the heat and mass correlations [19].

Defining the boundary conditions and considering over 30 inlet parameters, the total set of equations is solved using the MATLAB software 2020a. The boundary conditions are based on our previous study [8]; however, in this study, solar radiation is employed in the external radial boundary condition instead of *T_wall_*.

There are four boundary conditions: (1) the feed inlet is characterized by the inlet heat flux (Equation (9)); (2) at the feed outlet, the heat transfer with the exterior is zero, such that there is no energy source from the feed outlet side of the module toward the retentate (Equation (10)); (3) at the absorber shell interface, the boundary condition considers the absorbed solar energy (Equation (11)); and (4) at the center of the VMD module, there is no heat transfer from the center node (*n_r_*) to the previous node (*n_r_* − 1) (Equation (12)).

(9) $$Q_{x_{\left(r,0\right)}}=C_{p(r,0)}F_{\left(r,0\right)}T_{\left(r,0\right)}$$

(10) $$Q_{x_{\left(r,n_{x+1}\right)}}=0$$

(11) $$Q_{r_{\left(0,x\right)}}=G_{TA}{\varphi S}_{3\left(x\right)}$$

(12) $$Q_{r_{\left(n_{r},x\right)}}=0$$

**Figure 4:**
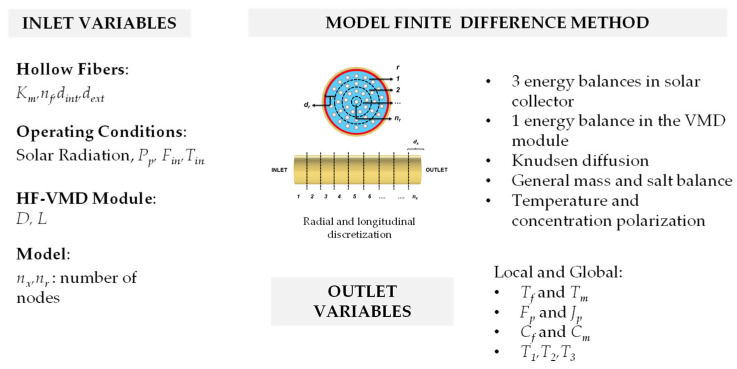
Inlet variables, equations, and outlet variables for the modeling of the HF-SC-VMD module.

With this model, it is possible to determine the local temperature in the external glass layer, internal glass layer, absorber, and retentate, as well as the permeate characteristics (temperature, flow rate, and salinity) and concentration near the membrane as Figure 4 summarizes it.

## 4. Energy Balances on the Integrated Solar Collector

The energy transfer through the HF-SC-VMD module needs to be described explicitly by the energy balances to quantify the amount of energy that the module receives, the amount of energy that can be employed for water vaporization within the shell tube, and the amount of energy lost. First, the solar irradiation is calculated considering the environmental conditions and the solar collector properties. Then, the energy balances over the absorber and the internal and external glass layers are successively described to determine the quantity of energy that the fluid receives for permeate generation and the amount of energy lost to the environment.

The main assumptions considered for the heat transfer through the solar collector are the following.

(1) The heat transfer mechanisms for each layer are:
  (a) External tube and environmental air: convection and radiation;
  (b) External and internal glass tubes: radiation;
  (c) Internal glass tube and shell: convection and radiation;
  (d) Shell and fluid at shell wall: convection.
(2) The heat transfer coefficient of the wind in the environmental air is assumed to be constant (10 W/m^2^ C).
(3) As a result of their thicknesses, there is no heat loss via conduction in the shell and glass tubes.
(4) The heat losses are considered over the entire surface area of the shell.

### 4.1. Absorbed Solar Radiation

**Figure 5:**
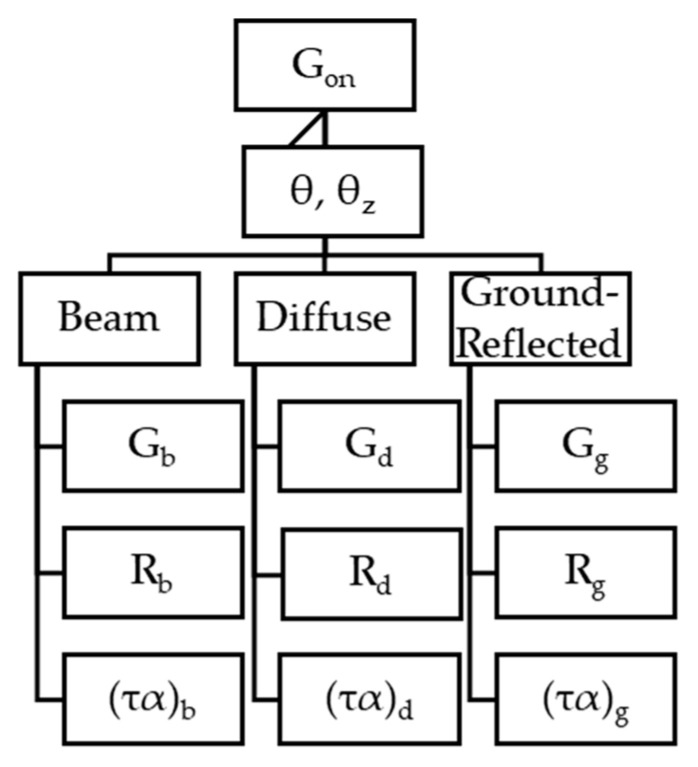
Calculation methodology for the absorbed solar radiation.

The first flow of energy comes from the incident solar radiation, which depends on the extraterrestrial solar radiation (*G_on_*), composed of the direct (*G_b_*), diffuse (*G_d_*), and ground-reflected (*G_g_*) radiation; the geometrical factors (*R_b_*, *R_d_*, and *R_g_*) for each type of radiation; and the incidence angle (θ and θ*_z_*). This energy is transmitted through the glass layers and finally absorbed by the absorber. The absorbed energy ($G_{TA}\varphi S_{3(x)}$) depends on the solar collector properties ((τα)*_b,d,g_*), that is, the emittance, transmissivity, and reflectance of the glass layers, as well as the absorptance of the absorber. The calculation methodology is summarized in the diagram shown in Figure 5.

The calculation of each term illustrated in Figure 5 is explained in detail in Ref. [20]. In general, these terms can be expressed as

(13) $$G_{TA}=G_{b}R_{b}{\left(\tau \alpha \right)}_{b}+G_{d}F_{cs}{\left(\tau \alpha \right)}_{d}+(G_{b}+G_{d})\rho_{g}F_{cg}{\left(\tau \alpha \right)}_{d}.$$

Based on the amount of absorbed energy, the next step is to estimate the energy transferred to the fluid, which is determined by the energy balance in the absorber and is described in detail in Section 4.2.

### 4.2. Absorber

**Figure 6:**
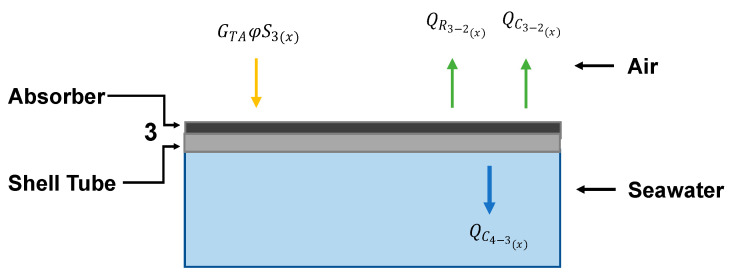
Energy flows at the absorber shell tube interface.

The absorber is one of the principal components of the solar collector. It absorbs the solar radiation ($G_{TA}$) that is transferred via convection to the fluid ($Q_{C_{4{-3}_{\left(x\right)}}}$). Part of this energy is lost and exchanged via natural convection ($Q_{C_{3-2_{\left(x\right)}}}$) and radiation ($Q_{R_{{3-2}_{\left(x\right)}}}$) toward the internal glass layer. An energy balance over the absorber is established to estimate the flow of the solar energy employed for water vaporization as shown in Figure 6.

The absorbed solar energy depends directly on the solar energy transmitted to the portion of the absorber exposed to solar radiation (φ), which is expressed as $G_{TA}{\varphi S}_{3\left(x\right)}$. The exposed surface area (${\varphi S}_{3\left(x\right)}$) is assumed to be half of the shell surface area, considering that the solar energy only arrives from the top and does not come into contact with the entire shell surface (*S_3_*).

The absorbed solar energy is also determined by the following properties of the absorber layer:

− The efficiency ($\eta_{rv}$) of a reflective surface ($S_{rv}$) to transmit the energy to the absorber. An efficiency of 80% is assumed here.
− The solar concentration factor (*CF*) determines the increase in the absorbed solar radiation. Here, the concentration factor is defined in Equation (14) as the ratio of the surface area that reflects and transmits the energy ($S_{rv}$) to the absorber surface area ($\varphi S_{3}$). A higher *CF* corresponds to a larger amount of absorbed solar power.
  (14) $$CF=\frac{S_{rv}}{{\varphi S}_{3}}$$
  (15) $$Solar\;power\;absorbed\;by\;the\;absorber\;=\;G_{TA}{\varphi S}_{3\left(x\right)}CF\eta_{rv}=G_{TA}S_{rv}\eta_{rv}$$

Conversely, the three other energy flows (convection, diffusion, and radiation) are considered over the entire shell surface. The energy balance considering heat transfer with the fluid and the exterior over the entire surface of the shell is described such that

(16) $$G_{TA}{\varphi S}_{3\left(x\right)\;}CF\eta_{rv}-Q_{C_{4-3\left(x\right)}}-Q_{R_{3-2\left(x\right)}}-Q_{C_{3-2}\left(x\right)}=0.$$

The solar energy is transferred from the absorber through the shell and finally to the fluid. Considering the relative thicknesses of the absorber and the tube (0.05 mm and 5.0 mm, respectively), the heat loss via conduction is neglected. Therefore, the temperature on the two sides of the shell is assumed to be constant and the convection in the feed shell is considered.

(17) $$Q_{C_{4-3\left(x\right)}}=h_{\left(4,x\right)}S_{3\left(x\right)}(T_{3(x)}-T_{4(x)})$$

The heat transfer coefficient depends on the fluid regime near the shell and is calculated for every longitudinal unit; the fluid regime depends on the fluid temperature near the shell ($T_{4(x)}$), which in turn depends on $T_{3(x)}$. $T_{3(x)}$ also varies longitudinally as a result of the influences of the absorbed energy and the fluid hydrodynamics. The heat losses toward the internal glass layer are defined by natural convection ($Q_{C_{4-3(x)}}$) and radiation ($Q_{R_{4-3(x)}}$) assuming that the space between the shell and the internal glass layer is filled with air.

(18) $$Q_{C_{3-2(x)}}=h_{c-3(x)}S_{3(x)}\left(T_{3(x)}-T_{2(x)}\right)$$

(19) $$Q_{R_{3-2(x)}}=h_{r-3(x)}S_{3(x)}\left(T_{3(x)}-T_{2(x)}\right)$$

The calculation of the convective and radiative heat transfer coefficients ($h_{c-3(x)}$ and $h_{r-3(x)}$, respectively), depends on the internal glass tube temperature ($T_{2(x)}$), shell temperature ($T_{3(x)}$), internal glass tube radius (*R*_2_), and shell radius (*R*_3_) [21,22].

(20) $$h_{c-3}=\frac{2k_{gf}}{2R_{3}ln\left(\frac{R_{2}}{R_{3}}\right)}$$

The effective thermal conductivity describes the natural convection with air ($k_{gf}$), which is related to the thermal conductivity ($k_{g}$) and kinetic viscosity ($k_{v}$) of air, as well as other air properties [22]. The calculation of this heat transfer coefficient is estimated via the Rayleigh (Ra) number, which accounts for the geometrical configuration and hydrodynamics [20,22].

(21) $$k_{gf}=k_{g}0.386\left({\left(\frac{Pr}{0.861+Pr}\right)}^{0.25}{\left(Ra_{c}\right)}^{0.25}\right)$$

(22) $$Pr=\frac{C_{p}\mu }{k}$$

(23) $${Ra}_{c}={\left(ln\left(\frac{R_{2}}{R_{3}}\right)\right)}^{4}\frac{{Ra}_{L}}{{\left(({2R_{2})}^{-0.6}+{\left(2R_{3}\right)}^{-0.6}\right)}^{5}}$$

(24) $$Ra_{L}=\frac{g\beta Pr\left(T_{3(x)}-T_{2(x)}\right)}{k_{v}^{2}}$$

Conversely, the heat losses transferred via radiation are described by the Stefan–Boltzmann law, which relates the energy that a surface emits to its temperature. The main material property is the emissivity (ε). For black surfaces, such as the absorber, the emissivity is elevated (ε_3_). Assuming that the emissivity is equal to the absorptivity (α ~ 0.9), most of the incident solar radiation absorbed by a surface will also be emitted. Glass tubes are employed here for this reason. The absorptivity of glass tubes at wavelengths higher than 3 μm is elevated, which ensures that a large part of the emitted infrared energy remains inside the solar collector. The radiation heat transfer coefficient describes this phenomenon ($h_{r-3}$), as shown in Equation (25) [20,21].

(25) $$h_{r-3}=\frac{\sigma ((T_{3(x)}^{2}-T_{2(x)}^{2})(T_{3(x)}+T_{2(x)})}{\frac{1}{\varepsilon_{3}}+\frac{R_{3}}{R_{2}}\left(\frac{1}{\varepsilon_{2}}-1\right)}$$

After describing the energy flows in the absorber, the heat transfers through the internal and external glass tubes are examined to determine the temperature at each point in the solar collector.

### 4.3. Internal Glass Tube

The function of the internal glass tube is to transmit solar energy and reduce heat losses to the environment. The high transmissivity of this material at solar radiation wavelengths and its low emittance in the infrared make glass an interesting material to reduce the amount of energy transferred to the environment.

When examining the internal glass layer, the prevailing consideration is that heat loss occurs primarily via radiation from inside the glass tube to outside the glass tube as a result of the vacuum-filled space between the two glass layers.

**Figure 7:**
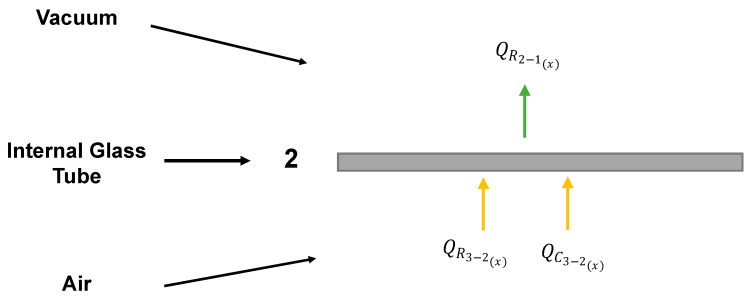
Energy flows with respect to the internal glass tube.

The energy balance for the internal glass tube involves the incoming energy via radiation and convection from the absorber to the internal glass layer and the outgoing energy via radiation from the internal glass layer to the external glass layer ($Q_{R_{{2-1}_{\left(x\right)}}}$) as illustrated in Figure 7.

(26) $$Q_{R_{3-2(x)}}+Q_{C_{3-2(x)}}-Q_{R_{2-1(x)}}=0$$

The radiative heat transfer coefficient between the internal and external glass tubes depends on the tube temperatures ($T_{1(x)}$ and $T_{2(x)}$, respectively), and their dimensions ($R_{1}$ and $R_{2}$, respectively) [22].

(27) $$h_{r-2(x)}=\frac{\sigma (T_{2(x)}^{2}+T_{1(x)}^{2})(T_{2(x)}+T_{1(x)})}{\frac{1}{\varepsilon_{2}}+\frac{R_{2}}{R_{1}}\left(\frac{1}{\varepsilon_{1}}-1\right)}$$

(28) $$Q_{R_{2-1(x)}}=h_{r-2(x)}S_{2(x)}\left(T_{2(x)}-T_{1(x)}\right)$$

Finally, an energy balance is established for the external glass tube in contact with the environmental air to determine the temperature at this point.

### 4.4. External Glass Tube

**Figure 8:**
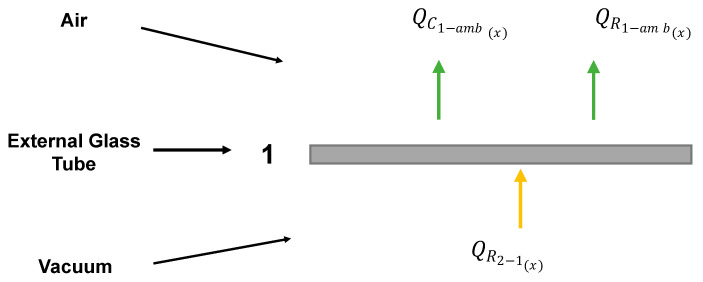
Energy flows with respect to the external glass tube.

The use of a vacuum, coupled with a second glass tube, reduces the heat losses of the solar collector. Nevertheless, there is still a heat exchange via convection and radiation with the environmental air. The energy balance for the temperature calculation at the external glass tube is represented in Figure 8.

(29) $$Q_{R_{2-1}(x)}-Q_{C_{1-amb}(x)}-Q_{R_{1-amb(x)}}=0$$

The convection term corresponds to the heat exchange resulting from wind. Here, a constant value is assumed (*h_w_* = 10 W/m^2^ C) [20].

(30) $$Q_{C_{1-amb}}=h_{w}S_{1(x)}\left(T_{1(x)}-T_{amb}\right)$$

For the radiative heat transfer coefficient, the following equations are employed [22]:

(31) $$h_{r-1}=\varepsilon_{1}\sigma \left(T_{1\left(x\right)}+T_{amb}\right)\left(T_{1\left(x\right)}^{2}+T_{amb}^{2}\right)\frac{\left(T_{1\left(x\right)}-T_{sky}\right)}{\left(T_{1\left(x\right)}-T_{amb}\right)},$$

(32) $$T_{sky}=0.0522\left(T_{amb}^{1.5}\right).$$

After describing the energy balance in each component of the solar collector, using Equations (16), (26) and (29), it is possible to calculate the temperature and energy flows at each layer by coupling them with the HF-VMD model.

The solar power absorbed by the absorber over the shell (first term in Equation (16)) is employed to assess the magnitude of the power required to achieve a certain average value of $\overset{=}{T_{wall}}$ and the production related to it.

With the full HF-SC-VMD model developed here, it is possible to determine the temperature profile not only in the membrane module but also in the solar collector and, most importantly, the amount of permeate flux produced for a given solar irradiation.

## 5. Simulation of the Influence of Solar Radiation on the Performance of a Semi-Industrial-Scale HF-SC-VMD Module

Considering the objective of developing an integrated HF-SC-VMD module for seawater desalination using solar energy as the principal energy provider, the influence of the absorbed solar energy on the performance of a given semi-industrial-scale module is discussed in this section.

Regarding solar radiation, two principal analyses were performed.

- The effects of the absorbed solar power on the permeate flux, flow rate, and bulk temperature at the module outlet were studied by varying *CF* to obtain average $\overset{̿}{T_{wall}}$ values of 50 °C, 60 °C, and 70 °C. Pure water was used to isolate the effect of the solar radiation. The module and operating conditions employed, except for the *T_wall_* value, are shown in Table 1 and Table 2.
- The temperature variation and permeate production were compared with the approach realized by Alfonso et al. [8], which considered a constant *T_wall_*, referred to in this study as the first approach. To make this comparison, pure water was employed and most parameters (e.g., the feed velocity, *F_in_*, HF-SC-VMD module dimensions, and *P_p_*) were kept the same for the two cases. The feed temperature was set to 40 °C (Reynolds number (Re): 637). The module characteristics and operating conditions are shown in Table 1 and Table 2.

Note that, in this study, in the so-called second approach, the temperature at the shell wall is not fixed nor is it constant along the module and, according to the heat exchange between the transferred solar radiation and the fluid temperature near the shell, the value of the temperature at this point may change, creating a longitudinal temperature profile at the shell wall. *T_wall_* is then calculated locally, and an average value is estimated between the module inlet and outlet at the shell surface ($\overset{̿}{T_{wall}}$) according to the absorbed solar power; this value is varied by increasing the concentration factor. The environmental, radiative, and optical conditions and parameters are shown in Table 3, Table 4, Table 5 and Table 6 based on Ref. [2]. The numbers of radial and longitudinal discretizations used in this study are 8 and 12, respectively, based on the computational time, feed temperature, and permeate flux variation with the mesh size.

### 5.1. General Performance

First, the power values required to achieve average temperatures, $\overset{̿}{T_{wall}}$, of 50 °C, 60 °C, and 70 °C at the shell wall were calculated considering the above information. These absorbed power values are listed in Table 7 with their respective permeate production flow rates, permeate fluxes, and outlet fluid temperatures. The results obtained using the first approach are also given in Table 7.

Table 7 shows the concentration factors required to attain the specified values of $\overset{̿}{T_{wall}}$, demonstrating the high requirement of enhancing the solar power directed to the module. For example, regarding the power requirements of such a system, achieving an average $\overset{̿}{T_{wall}}$ of 70 °C with a feed of 40 °C requires an absorbed solar power of 6.01 kW, demanding a solar collector with a very high solar concentration factor (*CF*) of 285.

**Figure 9:**
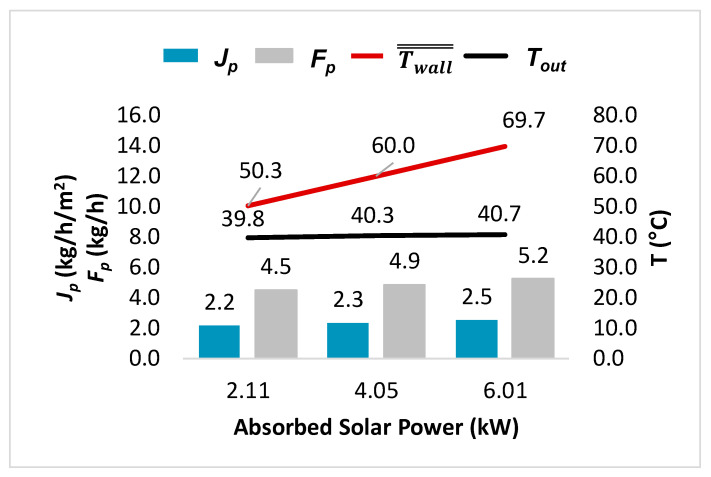
Performances of the HF-SC-VMD module at different absorbed solar powers (*v*: 0.5 m/s, *F_in_*: 3252 L/h, *T_in_*: 40 °C, Re: 637, *P_p_*: 6.0 kPa, *C*: 0 g/kg, ϴ: 53%, *D*: 3.5 cm, *L*: 42.5 cm, and *K_m_*: 3.84 × 10^−6^ s mol^0.5^/m/kg^0.5^).

Figure 9 illustrates the permeate flux, flow rate, $\overset{̿}{T_{wall}}$, and *T_out_* for the different absorbed power values. For an absorbed power of 2.11 kW, $\overset{̿}{T_{wall}}$ reaches 50.3 °C. An absorbed power increase to 6.0 kW generates a $\overset{̿}{T_{wall}}$ increase to 69.7 °C. This implies that an increase of 3.9 kW in the absorbed power will result in a $\overset{̿}{T_{wall}}$ increase of 19.4 °C. In general, we observe a linear relationship between the solar power and $\overset{̿}{T_{wall}}$, as shown in Figure 9.

### 5.2. Temperature Profiles

Longitudinally, the wall temperature exhibits increases of 1.23 °C, 2.74 °C, and 4.10 °C for $\overset{̿}{T_{wall}}$ values of 50 °C, 60 °C, and 70 °C, respectively, indicating that there is a higher variation in the longitudinal wall temperature with increasing absorbed solar power. This can be explained by the hypothesis that the heat loss occurs over the entire shell surface of the solar collector (constant) and by the fixed efficiency of the reflective surface ($\eta_{rv}:$ 0.8), in addition to other phenomena occurring during the VMD process, including a higher amount of solar energy employed for fluid heating as opposed to water vaporization. For a $\overset{̿}{T_{wall}}$ of 50 °C, 100% of the transferred power is employed for water vaporization, while for a $\overset{̿}{T_{wall}}$ of 70 °C, 58.3% of the transferred power is employed for permeate production and 41.3% is employed to increase the sensible energy of the fluid, as evidenced by the increase in the fluid temperature at the module outlet. The question that arises involves identifying the phenomena limiting the heat transfer of the absorbed energy to the fluid for permeate production.

Second, the bulk outlet temperature was determined for each value of $\overset{̿}{T_{wall}}$. In Figure 9, *T_out_* shows only a minor change in response to the solar power despite a 20 °C increase in $\overset{̿}{T_{wall}}$ (from 50.3 °C to 69.7 °C). This is primarily due to the transferred energy employed for permeate generation and the increase in the sensible energy. For a solar power of 2.11 kW, all the transferred energy is used for permeate production. A portion of the sensible energy needs to be allocated to provide sufficient power for the permeate flow generation, resulting in a slight decrease in the average outlet temperature to 39.8 °C. In the scenario with 6.01 kW of solar power, 58.3% of the transferred energy is utilized for water vaporization, while 41.7% contributes to increasing the sensible energy. This leads to a slight increase in the average outlet feed temperature, which reaches 40.7 °C. This shows the importance of considering not only the fluid temperature but also the energy flows to understand the phenomena occurring in the module.

**Figure 10:**
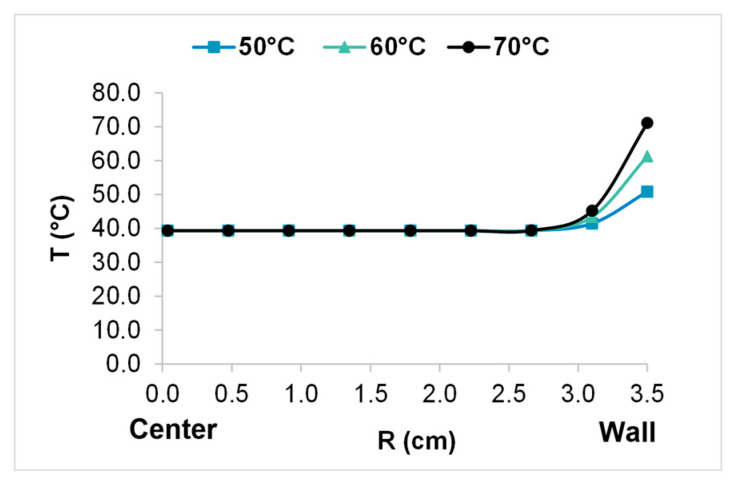
Radial temperature profile in the bulk feed for different values of $\overset{̿}{T_{wall}}$.

Under the tested conditions, the temperature polarization is negligible (TPC: 1.00 for Re: 637 at the feed inlet). The principal bulk temperature decrease occurs near the shell, where the fluid temperature and the number of fibers per layer are highest and therefore the permeate flow rate is highest. This is represented by the radial fluid temperature profile in the bulk feed, as shown in Figure 10, where *R* = 0 cm corresponds to the module center and *R* = 3.5 cm corresponds to the wall.

The radial temperature profile in the bulk feed enables an assessment of how the solar radiation is transferred and used for vaporization when traversing successive fiber layers from the periphery to the center under specific module, membrane, and operating conditions. Figure 10 indicates that, at a distance of 2.6 cm from the module center, the fluid temperature decreases to approximately the temperature of the feed at the module inlet, meaning that the permeate flux is constant from 2.6 cm to the module center and is proportional to the fluid sensible energy. In other words, this decrease occurs between the shell and a distance from the shell wall of 26%.

### 5.3. Permeate Production

The permeate flow obtained for the different solar powers ranged between 4.51 kg/h and 5.24 kg/h, as depicted in Figure 9, which means that an increase of 3.9 kW in the absorbed solar power produces an augmentation of 17% in the permeate flow rate. That is, for each additional kilowatt of solar heat provided, a 0.19 kg/h increase in the permeate flow rate is produced for this 2.1 m^2^ semi-industrial-scale module under the conditions studied. However, it might be possible to further enhance the module performance by modifying the operating conditions and the module geometry.

Another factor to consider is the permeate flux, which shows a proportional increase with the permeate flow. The permeate flux varies from 2.16 kg/h/m^2^ to 2.51 kg/h/m^2^ for absorbed solar powers of 2.11 kW and 6.01 kW, respectively, for the same membrane surface area. As for the permeate flow, an increase of 3.9 kW in the absorbed solar power produces an increase of 16% in the permeate flux. Based on this result, the question that arises concerns how to determine the point at which it becomes worthwhile to increase the solar energy concentration, considering that the permeate production is not significantly influenced by this energy input under the tested conditions.

Furthermore, increasing the absorbed solar radiation produces slight variations in the bulk temperature. This illustrates the efficiency of employing the solar power transferred to the fluid for permeate production and not for increasing the fluid sensible power. It is expected that $\overset{̿}{T_{wall}}$ will vary more than the bulk feed temperature, considering that, in the shell, the heat loss is not due to vaporization but to the exchange to the environment, which represents less than 1% of the absorbed power. In conclusion, the absorbed solar power required to produce a $\overset{̿}{T_{wall}}$ of 70 °C for the semi-industrial-scale module corresponds to 6.01 kW and a permeate production of 5.24 kg/h.

### 5.4. Comparison with the First Approach

To facilitate the comparison of the results obtained using the solar collector equations with those of the reference case with a fixed *T_wall_*, we examined a scenario in which $\overset{̿}{T_{wall}}$ is set to 69.7 °C. For this $\overset{̿}{T_{wall}}$, with the second approach, the permeate production is 0.78 kg/h higher than in the reference case (Table 8) for the same flow rate and module geometry. This is primarily due to the amount of transferred solar power.

The transferred power to the fluid corresponds to 2.48 kW in the first approach, as opposed to 5.99 kW in the second approach (Table 8). This difference explains the variations in the permeate flow rate and the outlet feed temperature. In the first approach, it was assumed that the fluid temperature near the shell is 70 °C and that the heat transfer takes place via conduction, resulting in a lower transferred power compared with the second approach. In the second approach, a $\overset{̿}{T_{wall}}$ of 70 °C over the shell surface does not represent the same amount of power because the heat transfer is via convection from the shell to the fluid. This means that the power transferred from a fluid with a temperature of 70 °C via conduction is different from the power transferred from a shell with a $\overset{̿}{T_{wall}}$ of 70 °C via convection.

Regarding the fluid temperature, the average bulk outlet temperature for the second approach is 40.7 °C in comparison with the reference case for which *T_out_* is 39.8 °C. A higher fluid temperature is achieved, considering that, for a fixed *T_wall_*, two factors may occur: first, the transferred solar power is not the same along the module length, and, second, ensuring a constant *T_wall_* of 70 °C means that there is a lower heat transfer toward the fluid, resulting in a lower permeate production.

**Figure 11:**
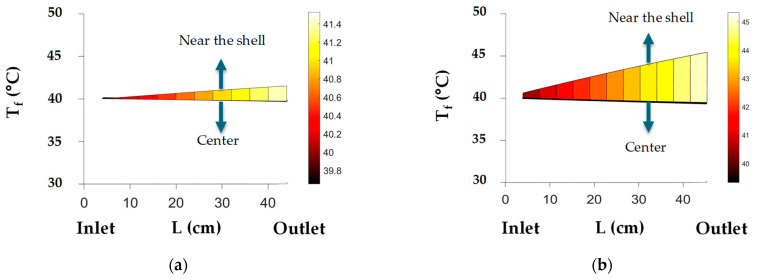
Longitudinal temperature profiles for the (**a**) 1st and (**b**) 2nd approaches.

Figure 11 shows the longitudinal temperature profiles near the shell and in the center of the module for the two approaches. These profiles reflect the energy transferred to the fluid and that is employed for water vaporization. In the first approach, all of the transferred power (2.48 kW) is employed for permeate generation (2.98 kW) and, as a consequence, the feed temperature near the shell does not show a large increase, as in the case of the second approach. Conversely, in the second approach, the transferred power is higher (5.99 kW) than the permeate power (3.49 kW), which represents 58% of the transferred power. This indicates that 42% of the power remains in the retentate, which explains the higher average bulk temperature in the fluid at the outlet in comparison with that at the entry (*T_in_*: 40.0 °C and *T_ou_*_t_: 40.7 °C), as well as the temperature near the wall.

This study shows the importance of considering the behavior of solar power and its impact on permeate production. The increase in the absorbed solar power led to the permeate flow rate being enhanced by 1.16 times for an absorbed power variation of 3.90 kW; this indicates that improvements need to be made in terms of the module dimensions and operating conditions.

Ultimately, by studying the principal causes of the heat losses and heat transfers over the full module, the HF-SC-VMD model integrating the description of the solar collector is a tool that can be employed to determine the maximum power that can be used without damaging the membranes, the power required to heat the fluid to a certain temperature, the achieved performance based on a determined solar power, and the optimum design of the solar collector.

## 6. Conclusions

The module concept studied here was based on a cylindrical bundle of VMD HFs (outside/in configuration) inside a shell surrounded by an integrated solar collector, with the assumption that only 50% of the solar collector area captures heat. In this module, the solar energy is supplied radially to vaporize water beginning from the external peripheric layers of the fibers and passing through different HF layers.

We developed a model allowing a full description of the HF-SC-VMD module when operated for desalination considering all the main phenomena (membrane distillation, temperature and concentration polarization, absorption of solar radiation, energy balances over the solar collector, radial and longitudinal heat and mass transfer, and seawater properties) and more than 30 inlet variables. This model estimates the amount of power required to reach a certain temperature at the shell wall and can be used to analyze and understand the impacts of the module design and operating parameters on the fluid temperature profiles and achievable module performances.

For a semi-industrial-scale module operating with pure water at 40 °C as a feed, this approach led to a permeate flow rate of 5.24 kg/h, representing an increase of 0.94 kg/h relative to the flow rate obtained using the previously published first approach. This higher estimation is primarily explained by the higher transferred thermal power to the fluid (second approach: 5.99 kW versus first approach: 2.5 kW).

In terms of the energy flows, the amounts of solar energy absorbed, transferred, and lost were estimated to evaluate the amount of energy employed for permeate production based on the module properties and operational conditions. The descriptions of the different phenomena in the module indicate the importance of understanding the energy flows in such systems.

Based on the simulations, we determined the following concerning the energy flows and management of an HF-SC-VMD module under the tested conditions.

− The heat losses remained lower than 0.54% of the absorbed power primarily because of the consistent surface area of the heat loss (the shell surface) and the fixed received surface efficiency (η*_rv_*: 0.80); therefore, it is important to consider the variation in the heat loss according to the receiver surface design and the method in which the energy is concentrated.
− Recovering the sensible energy from the concentrate back into the system is of interest and indicates some insights for full system designs.
− It is necessary to identify technical solutions to achieve the required concentration level for this first envisioned semi-industrial-scale module, which is not fully optimized to be compatible with the concept of an integrated solar collector HF-SC-VMD module. This suggests that the design and properties of the solar collector (absorption, transmission, and emissivity) should be optimized to minimize the reflective surface area while ensuring that the transferred thermal energy will be employed in its totality for permeate generation.
− Only the fibers located close to the shell wall are impacted by solar heating, having an enhanced production in comparison with a conventional module. At this stage, these results suggest that the module diameter and the bundle compacity will be important parameters; accordingly, their influences will be a topic of future research.

In future studies, module prototyping will allow the model to be validated and real performances to be tested; furthermore, the model of the HF-SC-VMD module will be integrated into a full system model to examine, via a parametric study, the influences of different variables and to optimize the module design.

## Figures and Tables

**Table 1:** Semi-industrial-scale hollow fiber solar collector vacuum membrane distillation (HF-SC-VMD) module characteristics.

Module Characteristics	Values
Number of fibers	2600
*d_ext_* (m)	10 × 10^−4^
*d_int_* (m)	6 × 10^−4^
*D* (m)	0.070
*L* (m)	0.425
*K_m_*_20°C_ (s∙mol^0.5^/m/kg^0.5^)	3.84 × 10^−6^

**Table 2:** Operating conditions for the semi-industrial-scale HF-SC-VMD module.

Operating Conditions	Values
*T_in_* (°C)	40
*v* (m/s)	0.5
*F_in_* (L/h)	3252
*T_wall_* (°C)	70
*P_p_* (Pa)	6000

**Table 3:** Environmental conditions [2].

Variable	Value
*n_d_* (number of the day)	228
Latitude (Φ) (°)	43.6
Slope horizon and plane (°)	0
Angle projected over azimuth surface (°)	0
Hour angle (°/h)	−15
Environmental temperature (°C)	20

**Table 4:** Beam radiation parameters [2].

Variable	Value
*r*_0_	0.97
*r*_1_	0.99
*r_k_*	1.02
Altitude	0.15

**Table 5:** Diffusion loss parameters [2].

Variable	Value
Extinction coefficient (1/m)	26.4
Number of glass tubes	2
Glass thickness (m)	0.0025
Ground reflectance ($\rho_{g})$	0.2

**Table 6:** Optical parameters [2,23,24,25].

Variable	Value
Absorptance of the absorber ($\propto_{ab})$	0.93
Emittance absorber ($\varepsilon_{ab})$	0.90
Refraction index glass (*n_g_*)	1.44
Glass emittance ($\varepsilon_{g})$	0.81
Glass reflectance ($\rho_{g}$)	0.11

**Table 7:** Influence of the absorbed solar power on the HF-SC-VMD module performance (*v*: 0.5 m/s, *F_in_*: 3252 L/h, *T_in_*: 40 °C, Re: 637, *P_p_*: 6.0 kPa, *C*: 0 g/kg, ϴ: 53%, *D*: 3.5 cm, *L*: 42.5 cm, and *K_m_*: 3.84 × 10^−6^ s mol^0.5^/m/kg^0.5^).

*CF*	Power (kW)	$\overset{̿}{T_{wall}}$ (°C)	*T_out_* (°C)	*F_p_* (kg/h)	*J_p_* (kg/h/m^2^)
100	2.11	50.3	39.81	4.51	2.16
192	4.05	60.0	40.27	4.86	2.33
285	6.01	69.7	40.73	5.24	2.51
-	1st approach with set *T_wall_*	70	39.79	4.46	2.14

**Table 8:** Energy flow comparison for the 1st and 2nd approaches.

Approach	1	2
*F_p_* (kg/h)	4.46	5.24
*Q_tr_* (kW)	2.48	5.99
*Q_v_* (kW)	2.98	3.49
∆*Q_f_* (kW)	−0.50	2.50

## Data Availability

The data supporting reported results are available from the corresponding author upon reasonable request. The data are not publicly available due to ongoing researches using a part of the data.

## Nomenclature

*C*	Salt concentration (g/kg)
*CF*	Solar concentration factor
CP	Concentration polarization
CPC	Concentration polarization coefficient
*D*	Module diameter (m)
*d_ext_*	External hollow fiber (HF) diameter (m)
*d_int_*	Internal HF diameter (m)
*d_r_*	Size of radial elementary unit (m)
*d_x_*	Size of longitudinal elementary unit (m)
*F*	Flow rate (kg/h) or (kg/s)
*F_in_*	Inlet flow rate (kg/h)
*F_out_*	Retentate flow rate at module outlet (kg/h) or (kg/s)
*F_p_*	Permeate flow rate (kg/h) or (kg/s)
*g*	Gravity (m^2^/s)
*G_b_*	Beam solar radiation (W/m^2^)
*G_d_*	Diffuse solar radiation (W/m^2^)
*G_g_*	Ground-reflected solar radiation (W/m^2^)
*G_on_*	Extraterrestrial solar radiation (W/m^2^)
*G_SC_*	Solar constant (1367 W/m^2^)
*G_TA_*	Total solar radiation absorbed per square meter of absorber surface (W/m^2^)
HF	Hollow fiber membrane
HF-SC-VMD	Hollow fiber solar collector vacuum membrane distillation module
HF-VMD	Hollow fiber vacuum membrane distillation module
*J_p_*	Permeate flux (kg/h/m^2^) or (kg/s/m^2^)
k	Fluid conductivity (W/m/K) or mass transfer coefficient (m/s)
$k_{gf}$	Heat transfer coefficient via natural convection with air
*K_m_*	Membrane permeability (s mol^0.5^/m/kg^0.5^)
*L*	Length (m)
MD	Membrane distillation
*n*	Number of nodes
*n_d_*	Number of the day
*n_f_*	Number of fibers
*n_g_*	Refraction index of glass
*n_r_*	Number of total radial nodes
*n_x_*	Number of total longitudinal nodes
Nu	Nusselt number
*P*	Pressure (Pa)
*P_p_*	Permeate pressure or vacuum pressure (Pa)
*P_sat_*	Saturation pressure (Pa)
*P_vp_*	Vacuum pump power (W)
*Q*	Power (W)
*Q_abs_*	Power absorbed from solar radiation (W)
*Q_p_*	Permeate power (W)
*Q_r_*	Radial heat power (W)
*Q_tr_*	Transferred solar power (W)
*Q_v_*	Vapor or permeate power (W)
Δ*Q_f_*	Global sensible energy flow change (W)
*r*_0*,*1*,k*_	Correction factors for climate types
*R*	Radius
Ra	Rayleigh number
Re	Reynolds number
RO	Reverse osmosis
*S*	Surface area (m^2^)
SC	Solar collector
SR	Solar radiation
*T*	Temperature (K) or (°C)
*T_in_*	Inlet temperature (K) or (°C)
*T_m_*	Temperature near the membrane (K) or (°C)
*T_out_*	Retentate temperature at module outlet (K) or (°C)
*T_wall_*	Temperature near the wall (K) or (°C)
$\overset{̿}{T_{wall}}$	Average wall temperature (K)
TP	Temperature polarization
TPC	Temperature polarization coefficient
*v*	Velocity (m/s)
VMD	Vacuum membrane distillation
**Subscripts**	
amb	Environment
b	Beam
C	Convection
d	Diffuse
f	Feed
g	Ground reflected or glass
in	Inlet
m	Membrane
out	Outlet
p	Permeate
r	Radial
(r, x)	Radial and longitudinal position
ref	Reference
rv	Reflective
sw	Seawater
SC	Solar collector
SE	Solar energy
v	Vacuum or vapor
x	Longitudinal
**Greek letters**	
α	Absorptance
β	Volumetric coefficient of expansion (1/K)
ε	Emittance
τ	Transmissivity
ρ	Reflectance, density (kg/m^3^)
ϴ	Packing density or incidence angle (°)
φ	Portion of shell tube covered by the absorber
η	Efficiency
σ	Stefan–Boltzmann coefficient (5.67 × 10^−8^ W/m^2^/K)
Φ	Latitude
